# The Significance of Secreted Phosphoprotein 1 in Multiple Human Cancers

**DOI:** 10.3389/fmolb.2020.565383

**Published:** 2020-11-24

**Authors:** Tengteng Wei, Guoshu Bi, Yunyi Bian, Suhong Ruan, Guangda Yuan, Hongya Xie, Mengnan Zhao, Rongming Shen, Yimeng Zhu, Qun Wang, Yong Yang, Donglin Zhu

**Affiliations:** ^1^Department of Thoracic Surgery, The Affiliated Suzhou Hospital of Nanjing Medical University, Suzhou Municipal Hospital, Suzhou, China; ^2^Department of Thoracic Surgery, Zhongshan Hospital, Fudan University, Shanghai, China; ^3^Department of Oncology, The Affiliated Suzhou Hospital of Nanjing Medical University, Suzhou Municipal Hospital, Suzhou, China

**Keywords:** SPP1, tumor prognosis, gene expression, miRNA, immune infiltration, biomarker, multiple human cancers

## Abstract

Malignant tumor represents a major reason for death in the world and its incidence is growing rapidly. Developing the tools for early diagnosis is possibly a promising way to offer diverse therapeutic options and promote the survival chance. Secreted phosphoprotein 1 (SPP1), also called Osteopontin (OPN), has been demonstrated overexpressed in many cancers. However, the specific role of SPP1 in prognosis, gene mutations, and changes in gene and miRNA expression in human cancers is unclear. In this report, we found SPP1 expression was higher in most of the human cancers. Based on Kaplan-Meier plotter and the PrognoScan database, we found high SPP1 expression was significantly correlated with poor survival in various cancers. Using a large dataset of colon adenocarcinoma (COAD), head and neck cancer (HNSC), lung adenocarcinoma (LUAD), and lung squamous cell carcinoma (LUSC) patients from the Gene Expression Omnibus (GEO) and The Cancer Genome Atlas (TCGA) databases, this study identified 22 common genes and 2 common miRNAs. GO, and KEGG paths analyses suggested that SPP1 correlated genes were mainly involved in positive regulation of immune cell activation and infiltration. SPP1-associated genes and miRNAs regulatory networks suggested that their interactions may play a role in the progression of four selected cancers. SPP1 showed significant positive correlation with the immunocyte and immune marker sets infiltrating degrees. All of these data provide strong evidence that SPP1 may promote tumor progress through interacting with carcinogenic genes and facilitating immune cells’ infiltration in COAD, HNSC, LUAD, and LUSC.

## Introduction

Malignant tumor represents the primary reason for deaths in the world and its incidence is growing rapidly (Bray et al., 2018). Lung cancer (LC), gastric cancer (GC), colorectal cancer (CRC), hepatocellular carcinoma (HCC), breast cancer (BC), cervical cancer, ovarian cancer, head and neck cancer (HNC), and endometrial cancer stand for the frequently occurring malignant tumors that kill millions of people every year (Wakelee et al., 2007; Botteri et al., 2008; Altekruse et al., 2009; Rocque and Cleary, 2013; Fan et al., 2014; Porceddu et al., 2015; Zhang et al., 2016; Urick and Bell, 2019). In the last several decades, the multimodal treatments have been developed, including surgery, radiotherapy, chemotherapy, together with molecular-targeted therapy, but the cancer prognosis is still poor (Tanaka and Arii, 2011; Zappa and Mousa, 2016; Shen et al., 2019; Schizas et al., 2020), which is partly because that effective diagnostic and prognostic markers are lacking.

Secreted phosphoprotein 1 (SPP1), also called Osteopontin (OPN), can be coded by human gene *SPP1* together with *Spp1* in murine. SPP1 is an integrin-binding glyco-phosphoprotein, which shows over-expression in a variety of tumors, such as liver cancer, LC, prostate cancer (PCa), BC, and CRC (Rangaswami et al., 2006; Blasberg et al., 2010; Kahles et al., 2014). SPP1 exerts its effects by interacting with receptors that ultimately lead to tumor progression, invasion, and metastasis (Denhardt et al., 2001; McAllister et al., 2008; Chae et al., 2009; Huang et al., 2017). Studies have demonstrated that high expression levels of SPP1 is associated with poor prognosis (Wei et al., 2018; Lamort et al., 2019). Moreover, SPP1 was involved in tumor immunosuppression and influenced the tumor microenvironment (Shinohara et al., 2008; Leavenworth et al., 2015; Shurin, 2018). Although SPP1 plays a role in many types of cancers, how its expression is regulated in relation to immune infiltration, gene mutation, gene and miRNA levels remains unclear.

The present work systematically assessed SPP1 level together with the correlation between SPP1 and prognosis in various human cancers. Moreover, the relationships of SPP1 with gene mutation and gene/miRNA expression were explored, and the common genes and their functions in four selected tumors were explored based on the Gene Expression Omnibus database (GEO) and The Cancer Genome Atlas (TCGA) databases. Then we investigated the influences of SPP1 on immune infiltration. This study provided a deep insight into the functions of SPP1 in human cancer, which may provide guidance for the development of promising therapies. We believe that SPP1 may play a greater role in future immunotherapy.

## Materials and Methods

### Oncomine Database Analysis

Oncomine database^1^, a cancer microarray database and web-based data mining platform (Saha et al., 2019), was used to analyze the expression level of the SPP1 gene in various types of cancers. The threshold was set as 0.001 for *P* value, 1.5 for fold change (FC), and gene ranking of all.

### Kaplan-Meier (K-M) Survival Analysis

We analyzed the overall survival (OS) and relapse-free survival (RFS) of different cancers patients by the use of Kaplan-Meier plotter^2^ (Hou et al., 2017). The GEO, TCGA, and EGA databases were used for K-M survival analysis. For determining the significance of each gene in predicting patient prognosis, all samples were divided as two groups based on the biomarker quantile expression. Then, the K-M survival plot was drawn to compare both groups; meanwhile, the hazard ratios (HRs) and the corresponding 95% confidence intervals (CIs) together with log-rank *P* values were determined.

### PrognoScan Database-Based Prognosis Analysis

PrognoScan^3^ collects tremendous public cancer microarray datasets and the clinical annotations, which has been used as the approach to assess the biological associations of gene expression levels with patient prognostic outcome (Mizuno et al., 2009; Pan et al., 2019). In the PrognoScan database, the minimal *P* value method is utilized to group cases to carry out survival analysis, so as to identify the optimum threshold for continuously measuring gene levels with no previous biological background. In this way, it allows to conduct systematic meta-analysis on several datasets. Sources for the databases come from GEO. The relationships of SPP1 gene levels with patient survival were examined in the diverse types of cancers. The cut-off point was set at Cox *P* value < 0.05.

### TIMER Analysis

The TIMER^4^ web server represents the integrated resource to systemically analyze the immune infiltrating levels among different types of cancers (Li et al., 2017). The “DiffExp” module was applied in examining SPP1 levels in cancer as well as matched non-carcinoma tissues in those TCGA^5^ tumors. The “Gene” module was adopted for exploring the correlation of SPP1 level with the immune infiltration among the four selected cancers. In TIMER, the deconvolution statistical approach reported in previous studies is utilized to deduce abundances of tumor-infiltration immune cells (TIICs) based on the gene expression patterns (Li et al., 2016). Immune infiltrates included CD4 + T cells, CD8 + T cells, B cells, dendritic cells (DCs), macrophages, and neutrophils. Scatterplots were drawn after the submitting SPP1, and statistical significance and partial Spearman correlation corrected by purity were subsequently shown. The “Correlation” module was used to plot the expression scatterplots between SPP1 and immune markers for a specific cancer. SPP1 was used to be a gene symbol on *x*-axis, and correlated marker genes were used as the gene symbols on the *y*-axis (Sousa and Määttä, 2016; Danaher et al., 2017; Siemers et al., 2017). The major immune markers in different immunocytes were observed from Table 1.

**TABLE 1:** Relationships of SPP1 with the immune cell marker sets based on TIMER.

Description	Gene markers	COAD		HNSC		LUAD		LUSC	
		cor	*p*	cor	*p*	cor	*p*	cor	*p*
CD8T + cells	CD8A	0.215	***	0.017	0.705	–0.051	0.262	0.089	0.051
	CD8B	0.132	*	0.03	0.513	–0.034	0.45	–0.026	0.578
T cells (general)	CD3D	0.104	0.036	–0.017	0.709	0.013	0.774	0.062	0.178
	CD3E	0.174	**	0.055	0.221	–0.092	0.041	0.034	0.457
B cells	CD19	–0.035	0.488	–0.056	0.214	–0.105	0.02	–0.011	0.803
	CD79A	–0.004	0.933	–0.036	0.425	–0.013	0.779	0.044	0.333
Monocytes	CD86	0.625	***	0.329	***	0.41	***	0.275	***
	CD115	0.544	***	0.374	***	0.365	***	0.281	***
TAMs	CCL2	0.541	***	0.265	***	0.326	***	0.209	***
	CD68	0.511	***	0.39	***	0.315	***	0.389	***
	IL10	0.444	***	0.186	***	0.236	***	0.204	***
M1 Macrophages	IRF5	0.244	***	0.256	***	0.189	***	0.276	***
	INOS	–0.174	**	0.128	*	–0.051	0.255	–0.144	*
	COX2	0.255	***	–0.069	0.129	0.099	0.027	–0.145	*
M2 Macrophages	CD163	0.651	***	0.469	***	0.303	***	0.344	***
	MS4A4A	0.635	***	0.5	***	0.334	***	0.384	***
	VSIG4	0.665	***	0.538	***	0.376	***	0.42	***
Neutrophils	CCR7	0.086	0.084	–0.007	0.885	–0.139	*	–0.05	0.277
	CD11b	0.687	***	0.457	***	0.34	***	0.212	***
Natural killer cells	KIR2DL1	0.178	**	0.04	0.378	–0.091	0.043	0.027	0.557
	KIR2DL3	0.189	**	0.02	0.651	–0.054	0.234	–0.037	0.417
DCs	HLA-DPA1	0.404	***	0.177	***	0.127	*	0.19	***
	HLA-DPB1	0.436	***	0.182	***	0.066	0.141	0.168	**
	HLA-DRA	0.397	***	0.181	***	0.179	***	0.223	***
	CD11c	0.644	***	0.481	***	0.198	***	0.157	**
	NRP1	0.617	***	0.339	***	0.136	*	0.251	***
Th1	STAT1	0.284	***	0.013	0.774	0.113	0.012	0.139	*
Th2	GATA3	0.227	***	0.057	0.21	–0.02	0.664	–0.189	***
	STAT6	–0.047	0.341	0.105	0.019	–0.223	***	–0.185	***
Tfh	BCL6	0.416	***	0.266	***	–0.011	0.808	–0.055	0.231
Th17	STAT3	0.175	**	0.094	0.038	–0.056	0.213	–0.059	0.195
Tregs	CCR8	0.358	***	0.208	***	0.124	*	0.115	0.012
	FOXP3	0.306	***	0.163	**	0.093	0.038	0.033	0.476
	STAT5B	0.099	0.047	0.249	***	–0.137	*	0.031	0.502
	TGFB1	0.532	***	0.193	***	0.189	***	0.073	0.112
T cell exhaustion	GZMB	0.022	0.666	–0.056	0.219	0.104	0.02	0.042	0.36
	HAVCR2	0.673	***	0.411	***	0.411	***	0.339	***
	(TIM-3)								
	PD-1	0.215	***	0.056	0.216	0.015	0.732	0.005	0.915

HNSC, head and neck cancer; COAD, colon adenocarcinoma; LUSC, lung squamous cell carcinoma; LUAD, lung adenocarcinoma; TAMs, tumor-associated macrophages; Tfh, Follicular helper T cells; Th, T helper cells; Treg, regulatory T cells; cor, R-values upon Spearman’s correlation. *P < 0.01; **P < 0.001; ***P < 0.0001.

### Correlated Gene Mutations and Copy Number Variation

Copy number variation (CNV) and gene mutation data were obtained using Xena browser from head and neck cancer (HNSC), colon adenocarcinoma (COAD), lung squamous cell carcinoma (LUSC), and lung adenocarcinoma (LUAD) cases. The detailed mutational data were obtained through MAF file by “Maftools” R/Bioconductor software (Mayakonda et al., 2018). Comparison of the distribution of gene mutations and CNV in different cancer was tested by Kruskal–Wallis test, where *p* value < 0.05 after adjustment for mutational frequency was considered significant. The “Maftools” oncoplot function was used to present results.

### Correlated Genes and MiRNA Analysis

Gene and miRNA expression data for COAD, HNSC, LUAD, and LUSC were obtained from TCGA database (Huang et al., 2019). These raw data were processed by background correction and normalization using the “affy” package (Song and Fu, 2019). The correlation between the expression of SPP1 and genes and miRNAs were evaluated in the R environment. The target miRNAs were predicted using miRWalk2.0^6^ (Gan et al., 2018). The common genes and miRNAs were validated by the Venn diagram^7^ (Huang et al., 2019). Subsequently, Gene Ontology (GO) functional annotation along with Kyoto Encyclopedia of Genes and Genomes (KEGG) pathway enrichment analyses were performed on the correlated genes by using “clusterProfiler” package at the thresholds of false discovery rate (FDR) < 0.05 and adjusted *p* < 0.05 (Kou et al., 2018).

### MiRNA-gene Regulatory Network Establishment

In line with interaction information of common miRNA and gene, the construction of the miRNA-gene regulatory network was performed using the R/networkD3 package.

### Correlated Genes in GEO

For data validation, the gene expression profiles of 139 COAD samples from GSE21510 and GSE110224, 1098 LUAD samples from GSE30219, GSE31210, GES3141, GSE37745, GSE50081, and GSE68465, 211 LUSC samples from GSE43580, GSE73403, and GSE67061 and 80 HNSC samples from GSE6631 and GSE13601 were obtained from the GEO data portal^8^ and analyzed using R language (Wang et al., 2017; Jin and Yang, 2019; Miao et al., 2019; Yang H. et al., 2019; Gao et al., 2020; Li et al., 2020; Qu et al., 2020). Patients in four cancers were divided into upregulated groups and downregulated groups base on the median expression of SPP1 (Zhao et al., 2018).

### The mRNAsi Calculation

The stemness index values of mRNA expression (mRNAsi) for all patients were calculated by “TCGAbiolinks” R package according to the mRNA levels by the machine learning algorithm of one-class logistic regression (OCLR) (Colaprico et al., 2016). *P* values < 0.05 were considered statistically significant.

### Relationships of SPP1 With the TIICs in CIBERSORT

This study adopted CIBERSORT^9^ to estimate the TIICs abundances based on gene levels in every case. All the gene expression profiles were collected from TCGA. The relationships of TIICs abundances with SPP1 level were evaluated in the R environment (Newman et al., 2015; Chen et al., 2019).

### Gene Correlation Analysis in GEPIA

The Gene Expression Profiling Interactive Analysis (GEPIA)^10^ is an interactive web serve established recently to analyze RNA sequencing data from 9,736 cancers as well as 8,587 non-carcinoma tissues in the Genotype-Tissue Expression (GTEx) and TCGA projects, according to the standardized processing method (Sun et al., 2019). This study investigated the relationship between the SPP1 and two receptors via GEPIA and adjusted by Spearman. SPP1 was shown in *x*-axis, whereas receptors in *y*-axis.

### PPI Networks

The Search Tool for the Retrieval of Interacting Genes/Proteins (STRING)^11^ provides experimental and predicted interactions among proteins (Kumar et al., 2019). STRING analyses were performed to analyze the protein-protein interaction (PPI) network, at the combined score >0.4 criterion.

### Statistical Analysis

Data obtained from TIMER, PrognoScan, and Kaplan-Meier plots were presented as HR and *P* values or Cox *P* values upon log-rank test. Meanwhile, the correlation strength between SPP1 and the immune cell was confirmed by the guidelines below: 0.00–0.19, “weak”; 0.20–0.29, “moderate”; 0.30–0.50, “strong”; >0.50, “very strong” (absolute value for all). A difference of *P* < 0.05 indicated statistical significance.

## Results

### SPP1 mRNA Level

**FIGURE 1:**
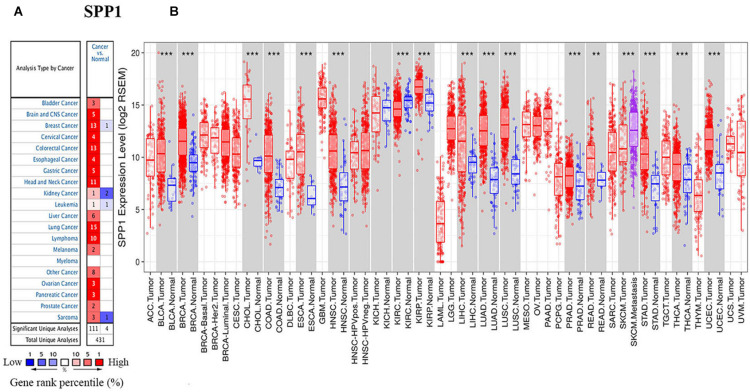
SPP1 levels in diverse cancer types and normal tissues. **(A)** High (red) or low (blue) SPP1 levels in various cancer types relative to non-carcinoma tissue samples based on the Oncomine database. **(B)** SPP1 expression in diverse cancers relative to the non-carcinoma tissue samples based on TCGA database was studied through TIMER. BLCA, bladder urothelial carcinoma; ACC, adrenocortical carcinoma; BRCA, breast invasive carcinoma; CHOL, cholangiocarcinoma; CESC, cervical squamous cell carcinoma and endocervical adenocarcinoma; ESCA, esophageal carcinoma; COAD, colon adenocarcinoma; HNSC, head and neck cancer; GBM, glioblastoma multiforme; KIRC, kidney renal clear cell carcinoma; KICH, kidney Chromophobe; LIHC, liver hepatocellular carcinoma; KIRP, kidney renal papillary cell carcinoma; LUSC, lung squamous cell carcinoma; LUAD, lung adenocarcinoma; PAAD, pancreatic adenocarcinoma; OV, ovarian serous cystadenocarcinoma; READ, rectum adenocarcinoma; PRAD, prostate adenocarcinoma; THCA, thyroid carcinoma; STAD, stomach adenocarcinoma; UCEC, uterine corpus endometrial carcinoma; THYM, thymoma; UVM, uveal Melanoma; and UCS, uterine carcinosarcomas (**P < 0.01, ***P < 0.001).

To investigate SPP1 expression, the Oncomine database was used to comparatively analyze mRNA levels across different tumor and non-carcinoma tissue samples. As a result, SPP1 showed higher expression in brain cancer, bladder cancer, cervical cancer, CRC, esophageal carcinoma (ESCA), GC, HNC, kidney cancer, liver cancer, LC, lymphoma, melanoma, PCa and pancreatic cancer in comparison with non-carcinoma tissues. The decreased SPP1 levers were observed in BC, kidney cancer, sarcoma and leukemia (Figure 1A).

We also used TIMER to study the expression of SPP1. The different SPP1 expression in cancer compared with non-carcinoma tissues was found by the box plots (Figure 1B), and the different expression levels were statistically significant upon Wilcoxon test. The SPP1 level apparently increased in cholangiocarcinoma (CHOL), breast invasive carcinoma (BRCA), bladder urothelial carcinoma (BLCA), COAD, ESCA, HNSC, liver hepatocellular carcinoma (LIHC), kidney renal papillary cell carcinoma (KIRP), LUSC, LUAD, rectum adenocarcinoma (READ), prostate adenocarcinoma (PRAD), thyroid carcinoma (THCA), uterine corpus endometrial carcinoma (UCEC), and stomach adenocarcinoma (STAD), in comparison with the non-carcinoma samples. The lower SPP1 levels only appeared in the kidney renal clear cell carcinoma (KIRC) relative to the non-carcinoma samples. The high expression of SPP1 in a variety of cancers suggested that it was related to clinical prognostic outcome.

### Prediction Significance of SPP1 in Cancers

**FIGURE 2:**
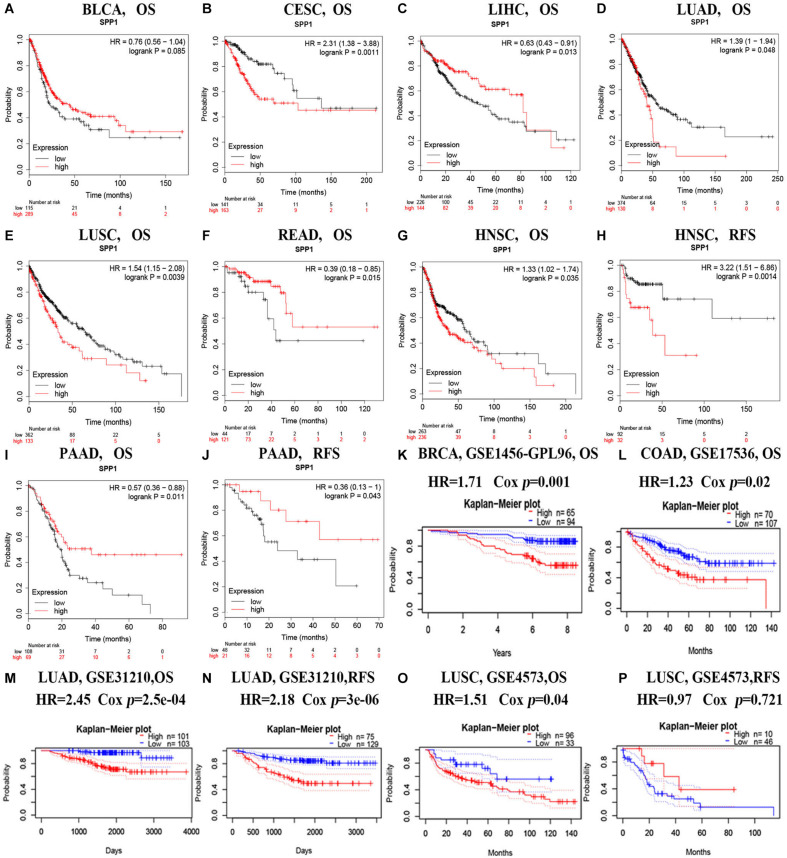
Survival for diverse cancer types according to SPP1 level based on the Kaplan-Meier plotter **(A–J)** and PrognoScan databases **(K–P)**. **(A–J)** The OS and RFS curves for BLCA (*n* = 404), CESC (*n* = 304), LIHC (*n* = 370), LUAD (*n* = 504), LUSC (*n* = 495), READ (*n* = 165), HNSC (*n* = 499 and *n* = 124) and PAAD cancer (*n* = 177 and *n* = 69). **(K,L)** Survival curves of OS in BRCA [GSE1456 (*n* = 159)] and COAD [GSE17536 (*n* = 177)]. **(M,N)** Survival curves of OS and RFS in LUAD [GSE31210 (*n* = 204) and GSE31210 (*n* = 204)]. **(O,P)** Survival curves of OS and DFS in LUSC [GSE4573, (*n* = 129) and GSE4573 (*n* = 56)]. RFS, relapse-free survival; OS, overall survival.

Subsequently, this study examined the SPP1 prediction significance for different cancer types based on the K-M plotter. Obviously, high SPP1 levels were significantly related to the dismal prognosis for several type cancers, including LUAD (OS HR = 1.39, 95% CI = 1-1.94, Cox *P* = 0.048), cervical squamous cell carcinoma (CESC) (OS HR = 2.31, 95% CI = 1.38-3.88, Cox *P* = 0.0011), HNSC (OS HR = 1.33, 95% CI = 1.02-1.74, Cox *P* = 0.035; RFS HR = 3.22, 95% CI = 1.51-6.86, Cox *P* = 0.0014), and LUSC (OS HR = 1.54, 95% CI = 1.15-2.08, Cox *P* = 0.0039) (Figures 2B,D,E,G,H). Contrastingly, high SPP1 expression showed better prognosis in READ (OS HR = 0.39, 95% CI = 0.18-0.85, Cox *P* = 0.015), LIHC (OS HR = 0.63, 95% CI = 0.43-0.91, Cox *P* = 0.013), and pancreatic ductal adenocarcinoma (PAAD) (OS HR = 0.57, 95% CI = 0.38-0.88, Cox *P* = 0.011; RFS HR = 0.38, 95% CI = 0.13-1, Cox *P* = 0.043) (Figures 2C,F,I,J). Differences in SPP1 level and prognostic outcome for BLCA were not significant (Figure 2A). These results identified SPP1 as an independent predictive factor in diverse types of cancer.

For better determining the SPP1 prediction ability for diverse cancer types, PrognoScan was adopted for evaluating how SPP1 level affected OS and RFS (Figures 2K–P). PrognoScan survival analysis revealed that SPP1 expression level was inversely related to the OS for BRCA (OS HR = 1.71, 95% CI = 1.23-2.38, Cox *P* = 0.001), COAD (OS HR = 1.23, 95% CI = 1.03-1.46, Cox *P* = 0.02), LUAD (OS HR = 2.45, 95% CI = 1.52-3.94, Cox *P* = 2.5e-04) as well as LUSC patients (OS HR = 1.51, 95% CI = 1.02-2.25, Cox *P* = 0.04). We also found patients with high SPP1 expression has a poor RFS in LUAD (RFS HR = 2.18, 95% CI = 1.57-3.02, Cox *P* = 3e-06) rather than in LUSC (RFS HR = 0.97, 95% CI = 0.82-1.14, Cox *P* = 0.721), this phenomenon was possibly resulted from the few LUSC patients. These results suggested that the increased SPP1 level was related to the dismal prognosis for BRCA, CESC, COAD, HNSC, LUAD, and LUSC cases.

### Correlated Genomic Alterations and Gene Expression

**FIGURE 3:**
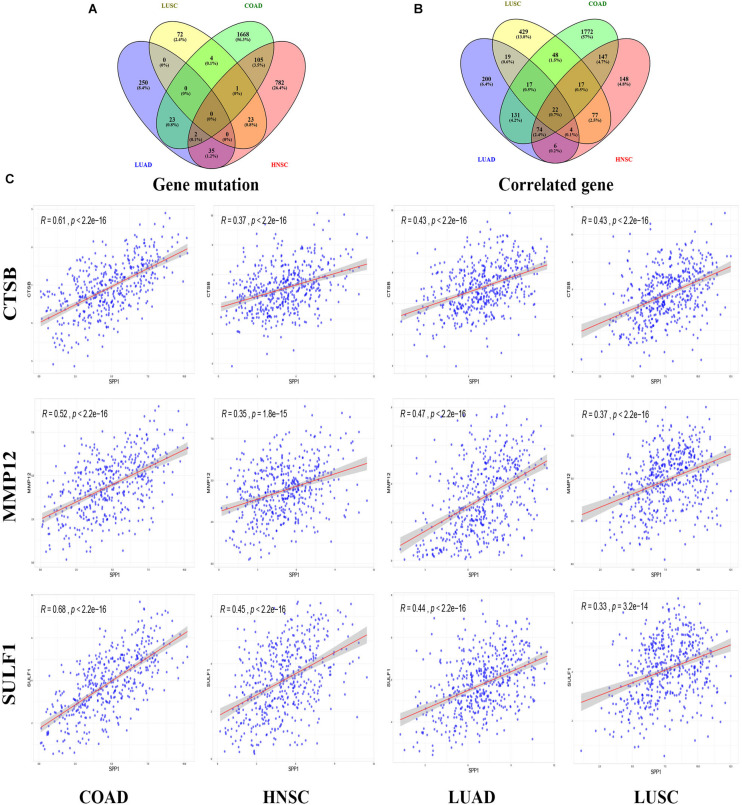
SPP1 correlated genomic alterations and genes in TCGA. Venn diagram depicting the distribution of SPP1 correlated genomic alterations **(A)** and genes **(B)** in COAD, HNSC, LUAD, and LUSC. **(C)** Correlation between SPP1 and CTSB, MMP12, and SULF1. HNSC, head and neck cancer; COAD, colon adenocarcinoma; LUSC, lung squamous cell carcinoma; LUAD, lung adenocarcinoma.

According to the screening criteria of consistent expression of SPP1 in different databases and sufficient number of cases, we found consistent prognostic correlations between SPP1 expression in COAD, HNSC, LUAD, and LUSC. For elucidating the possible mechanism of action by which SPP1 affected tumor prognosis, we investigated and identified correlated somatic mutations, CNV, and genes expression in four selected cancers. First, we divided somatic mutations and CNV into upregulated and downregulated groups base on the median expression of SPP1, and then investigated their distribution patterns. In the SPP1 hyperexpression group, 1803 SPP1-correlated mutate genes were identified in COAD, 948 in HNSC, 310 in LUAD, and 100 in LUSC, respectively (*p* value < 0.05) (Figure 3A). However, only three common mutant genes, PLD5, DTX4, and USP25 were statistically significant when compared with the group of low SPP1 expression (*p* < 0.05). In addition, in more than three types of cancer, the group with low SPP1 expression had no common mutated gene (data unshow). Then we conducted an integrated analysis for identifying the shared genes among the four cancers. Based on sequencing data mining, 2228 SPP1-correlated genes were identified in COAD, 495 in HNSC, 473 in LUAD and 633 in LUSC, respectively (*p* < 0.05 and | r| > 0.3) (Figure 3B). A total of 134 common correlated genes were identified in three or more cancers used in a Venn diagram–based approach. 22 genes (CTSB, LAPTM5, CALU, FCER1G, FCGR3A, VSIG4, NCF2, CD163, FCGR2A, SULF1, HCK, C3AR1, MS4A4A, MSR1, MMP12, HAVCR2, PIK3AP1, MSC, FCGR1A, CLEC5A, SIGLEC9, and ADAM12) were shared among all cases. Altogether 40 (22 + 17) genes were shared among COAD, HNSC, as well as LUSC; 40 (22 + 17) genes are common to COAD, LUAD, and LUSC; 97 (22 + 74) genes in COAD, HNSC, and LUAD; 26 (22 + 4) genes in HNSC, LUAD, and LUSC. By accessing TCGA data, we found all of 22 common genes were significant correlated with SPP1 expression and selected 3 genes (CTSB, MMP12, and SULF1) to be shown in scatter plot (Figure 3C).

**FIGURE 4:**
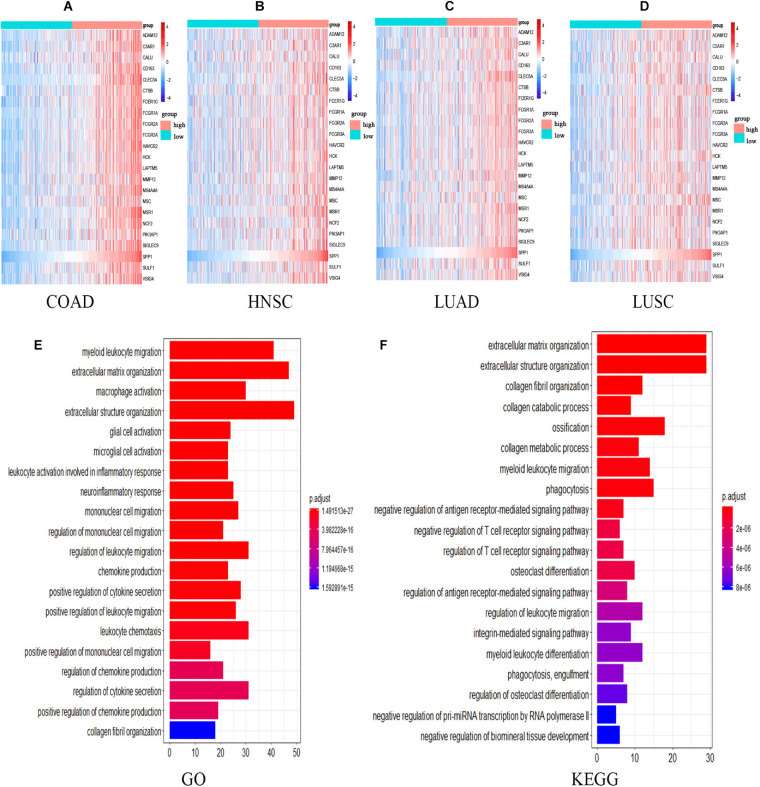
The expression and functional analyses of common genes based on TCGA. **(A–D)** Heatmap of SPP1 and 22 common genes expression in four types of cancers. Patients in four cancers were classified as high (red) or low (green) SPP1 group based on the median expression of SPP1. The gradual change from blue to red represents a gradual increase in gene expression. GO **(E)** as well as KEGG **(F)** analysis for the 134 common genes. HNSC, head and neck cancer; COAD, colon adenocarcinoma; LUSC, lung squamous cell carcinoma, and LUAD, lung adenocarcinoma.

**FIGURE 5:**
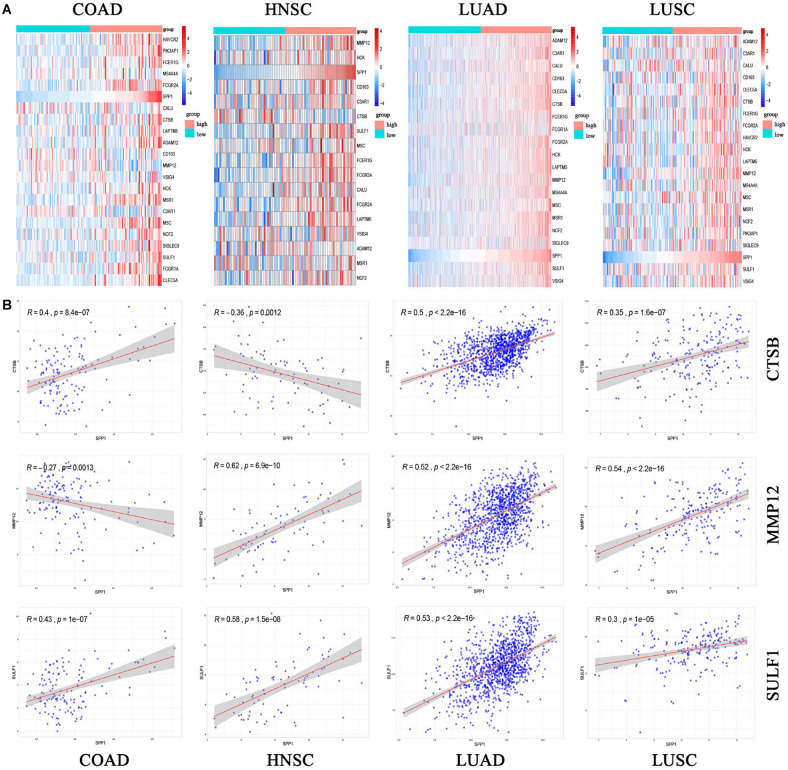
The common gene expression **(A)** and the correlation between SPP1 and CTSB, MMP12 and SULF1 in GEO **(B)**. High (red) or low (green) SPP1 expression group. The gradual change from blue to red represents a gradual increase in gene expression. HNSC, head and neck cancer; COAD, colon adenocarcinoma; LUSC, lung squamous cell carcinoma; and LUAD, lung adenocarcinoma. *P* < 0.05.

The expression of 22 common genes and SPP1 were classified as high or low SPP1 expression group in four types of cancer and showed in the heatmap (Figures 4A–D). Patients in four cancers were classified as high (red) or low (green) SPP1 expression group based on the median expression of SPP1. It is obvious that all of the 22 common genes levels showed markedly positive correlation with SPP1 expression. GO, and KEGG analyses were conducted to investigate the general functions of 134 common genes (Figures 4E,F). In the biological process (BP), these genes were mainly enriched into the myeloid leukocyte, organization of extracellular structure and matrix, and mononuclear migration, leukocyte, and macrophage activation and positive regulation of leukocyte and mononuclear migration. In KEGG analysis, the most enriched gene terms were extracellular matrix organization, extracellular structure organization, ossification, phagocytosis, and myeloid leukocyte migration. Based on the above results, these genes took part in the positive regulation of immune infiltration and may exert a vital part in cancer development and invasion. To conduct some validation of sorts, we further studied the expression of 22 common genes and their correlation with SPP1 in several GEO datasets. Consequently, most of 22 genes were more highly expressed in SPP1 high group than in SPP1 low group and 17 genes expressed in COAD, 12 in HNSC, 19 in LUAD, and 15 in LUSC, respectively (Figure 5A). The correlations between the expression of common genes and SPP1 were similar to the results based on TCGA. These genes were significantly positively associated with SPP1 expression, while only some genes, such as MMP12 in COAD and CTSB in HNSC, were negatively correlated (Figure 5B).

### Correlated miRNA Expression

**FIGURE 6:**
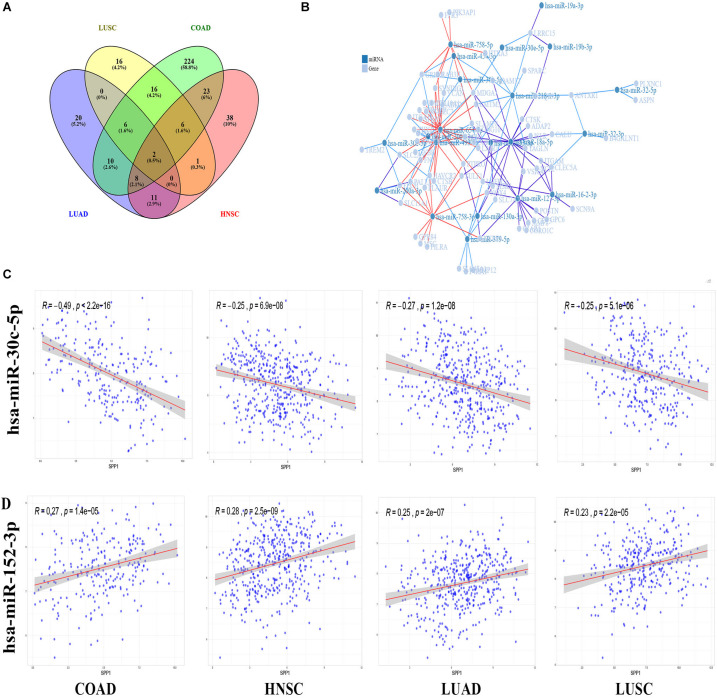
SPP1 correlated miRNA in TCGA and miRNA-gene regulatory network. **(A)** Venn diagram analysis of SPP1 correlated miRNA in COAD, HNSC, LUAD, LUSC. **(B)** The miRNA-gene regulatory network consists of 21 miRNAs and 71 genes. **(C)** The correlation between SPP1 and two common miRNAs. HNSC, head and neck cancer; COAD, colon adenocarcinoma; LUSC, lung squamous cell carcinoma; and LUAD, lung adenocarcinoma.

Next, the relationship of SPP1 level with miRNAs was examined based on data obtained from TCGA database. We identified 295 SPP1-correlated miRNAs were in COAD, 89 in HNSC, 57 in LUAD, and 47 in LUSC, respectively (|r| > 0.2, *p* value < 0.05) (Figure 6A). Two kinds of miRNAs are common to all. The correlation between SPP1 expression and two common miRNAs (hsa-miR-152-3p as well as hsa-miR-30c-5p) was shown in Figure 6C. This study identified altogether 19 common miRNAs to three cancers. The relationships between common miRNAs and correlated genes were obtained from miRWalk. Of the 134 genes, we identified 71 genes could interact with 21 common miRNAs. Then we constructed the miRNA-gene regulatory network to visualized their interaction (Figure 6B). The miRNA-gene regulatory network was composed of 92 nodes and 149 interactions. According to network, every 9 miRNAs (hsa-miR-16-2-3p, hsa-miR-127-3p, hsa-miR-18a-5p, hsa-miR-379-5p, hsa-miR-218-1-3p, hsa-miR-758-3p, hsa-miR-493-5p, hsa-miR-758-5p and hsa-miR-654-5p) had more than 5 interactions with common genes and every 20 genes (NOX4, CLEC5A, NXPE3, PTPRO, LHFPL2, MDGA1, ADAM12, LRRC15, FECH, GREM1, CTSB, ADD2, ANTXR1, LAIR1, MRO, PALLD, SLAMF8, SLC11A1, SULF1, SYNDIG1, and TAGLN) had more than 3 interactions with miRNAs. It is interesting that many of the 71 genes were suggested to be involved in tumor development. Based on the high connectivity, these SPP1 correlated miRNAs were very likely to interact with target genes and function in the progression of COAD, HNSC, LUAD, and LUSC.

### SPP1 Expression Was Correlated With mRNAsi and Immune Infiltration

**FIGURE 7:**
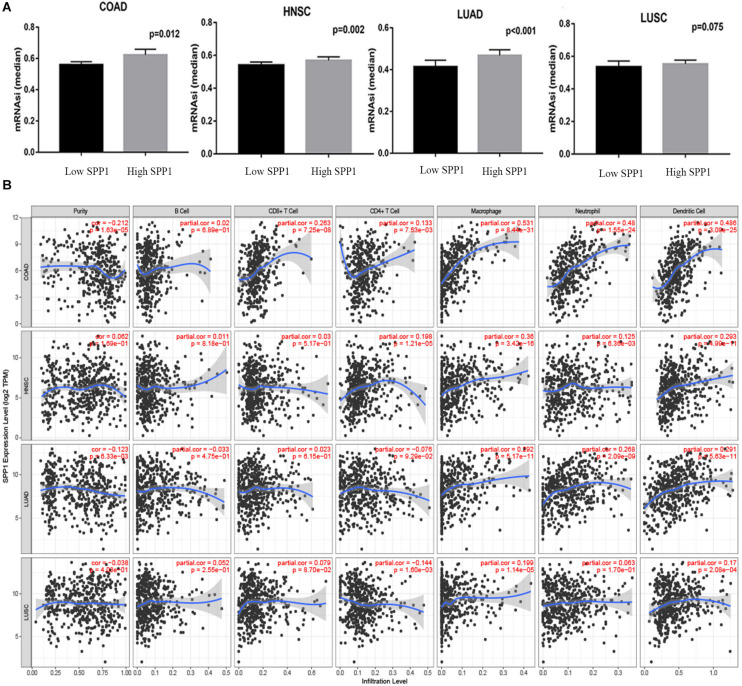
Relationship between SPP1 level and the mRNAsi and immune infiltrating degrees of COAD, HNSC, LUSC and LUAD. **(A)** The comparison of mRNAsi in high versus low SPP1 expression groups of different tumors. **(B)** Relationship between SPP1 levels and the immune infiltrating degrees of four selected cancer types. mRNAsi, mRNA expression based-stemness index; HNSC, head and neck cancer (*n* = 457); COAD, colon adenocarcinoma (*n* = 457); LUSC, lung squamous cell carcinoma (*n* = 507); LUAD, lung adenocarcinoma (*n* = 515).

Malta et al. (2018) reported mRNAsi as the efficient approach for evaluating the cancer differentiation level. The higher values for mRNAsi were associated with greater tumor dedifferentiation, as reflected in histopathological grade. As shown in Figure 7A, in COAD, HNSC, and LUAD, compared with low SPP1 expression group, the mRNAsi in high SPP1 group increased. However, difference was not significant in LUSC between two groups. Since immunocyte infiltration is the marker predicting cancer diagnosis and prognosis (Gentles et al., 2015). The 22 common genes identified in our report were mainly involved in the positive regulation of immune infiltration. Next, we used TIMER to investigate whether SPP1 level was related to the immunocyte infiltrating levels among four selected cancers (Figure 7B). In COAD, SPP1 level showed significant correlation with the macrophage (*r* = 0.531, *P* = 8.44e-31), neutrophil (*r* = 0.48, *P* = 1.55e-24), and DC (*r* = 0.486, *P* = 3.09e-25) infiltrating degrees. SPP1 expression level was also markedly associated with macrophages infiltrating (*r* = 0.36, *P* = 3.42e-16) in HNSC. In LUAD, the associations with macrophage (*r* = 0.292, *P* = 5.17e-11), neutrophil (*r* = 0.268, *P* = 2.09e-09), and DC (*r* = 0.291, *P* = 5.63e-11) infiltrating degrees were moderately positive. SPP1 expression was weakly related to tumor purity together with the CD4 + T cell, macrophage, and DC infiltrating degrees in LUSC. Additionally, using CIBER algorithm, the TIICs abundances and their correlation with SPP1 were assessed in four selected cancers based on gene expression profiles from TCGA. It was suggested that, the abundance of tumor infiltration of B memory cells and activated DCs was negatively correlated with SPP1 in COAD, HNSC, and LUSC. M0 macrophages tumor infiltration was positively correlated with SPP1 in COAD, HNSC, and LUAD. M2 macrophages tumor infiltration was positively correlated with SPP1 in four selected cancers (Supplementary Figure S3). These results potently suggested the vital part of SPP1 in immunocyte infiltrating degree, particularly for the macrophage, neutrophil, and DC infiltrating levels.

### Relationship Between SPP1 Levels With the Immune Cell Marker Sets

For further analyzing correlations of SPP1 expression with multiply TIICs, this study investigated relationship of SPP1 with a variety of immune marker sets in COAD, HNSC, LUAD, and LUSC via TIMER databases and adjusted by purity (Table 1). We found that SPP1 expression was positively correlated to TAMs markers (IL10, CD68, and CCL2), monocyte markers (CD115 and CD86), neutrophils marker (CD11b), M2 macrophage markers (CD163, MS4A4A, and VSIG4), T cell marker (HAVCR2), and DCs markers (NRP1 and CD11c). The above findings confirmed that SPP1 was specifically correlated with TIICs in COAD, HNSC, LUAD, and LUSC, thus implying the important part of SPP1 in the immune infiltrating and cancer microenvironment.

### SPP1 Was Positively Correlated With CD44 and ITGB1

**FIGURE 8:**
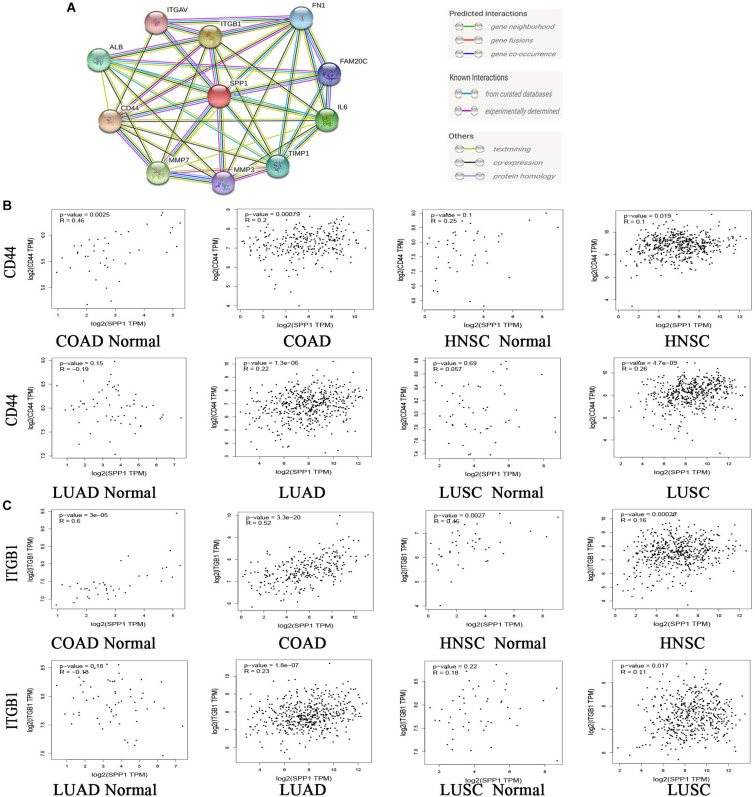
Correlation of SPP1 with receptors. **(A)** Protein-protein interaction (PPI) network. 10 molecules with the highest correlation with SPP1. **(B,C)** Correlation analysis between SPP1 and CD44 and ITGB1 in COAD, HNSC, LUAD, and LUSC from GEPIA. COAD, colon adenocarcinoma (*n* = 275), COAD normal (*n* = 349); HNSC, head and neck cancer (*n* = 519), HNSC normal (*n* = 44); LUAD, lung adenocarcinoma (*n* = 483), LUAD normal (*n* = 347); LUSC, lung squamous cell carcinoma (*n* = 486), LUSC normal (*n* = 338).

Secreted phosphoprotein 1 exerts its effects through interaction with receptors. A PPI network was constructed to understand the interaction between SPP1 and receptors (Figure 8A). The PPI network consisted of 11 nodes and 44 interactions. CD44 and ITGB1 are most closely related to SPP1. It has been reported that SPP1/CD44 signaling in the glioma perivascular niche promotes aggressive tumor growth (Wei et al., 2019), and ITGB1 was related to the dismal OS in NSCLC (Zheng et al., 2016). To ascertain whether SPP1 has a correlation with ITGB1 and CD44 in COAD, HNSC, LUAD, and LUSC. We investigated the relationship between SPP1 and two receptors via GEPIA (Figures 8B,C). In tumor tissue, SPP1 showed positive correlation with CD44 in LUAD (*R* = 0.22, *P* = 1.3e-06), COAD (*R* = 0.2, *P* = 0.00079), LUSC (*R* = 0.26, *P* = 4.7e-09), and HNSC (*R* = 0.1, *P* = 0.019), and was also associated with ITGB1 in COAD (*R* = 0.52, *P* = 3.3e-20), HNSC (*R* = 0.16, *P* = 0.00028), LUSC (*R* = 0.11, *P* = 0.017), and LUAD (*R* = 0.23, *P* = 1.8e-07). However, in adjacent normal tissue, SPP1 was only correlated with CD44 in COAD (*R* = 0.46, *P* = 0.0025) as well as ITGB1 in COAD (*R* = 0.6, *P* = 3e-05) and HNSC (*R* = 0.45, *P* = 0.0027). These data suggested that the interaction between SPP1 and CD44 and ITGB1 play a role in tumor progression in four selected types of cancer.

## Discussion

Malignant tumor represents a severe disorder that threatens human health, which becomes the primary public health issue (Smith et al., 2017). Exploring an effective cancer biomarker and detecting the related underlying pathways enriched by common miRNAs and genes are important to develop precision medicine and individualized treatment to manage cancer.

The present work suggested that the up-regulated SPP1 level was related to the dismal prognostic outcome in virous malignant tumors. In this work, 4 selected cancer databases were mined, which suggested that SPP1-correlated common genes and miRNAs may be involved in tumor immune infiltration, cancer progression and metastasis in COAD, HNSC, LUAD, and LUSC. The high SPP1 expression facilitated the infiltration level of immune cells and their markers, further confirming the interactions between SPP1 and common genes and miRNA in cancers. Collectively, such results suggested the vital part of SPP1 and its potential as a predictor in the regulation of immune infiltration, tumor prognosis, progression and metastasis.

Combined with the analysis results of Oncomine database and TIMER database, this study discovered the high expression of SPP1 in the bladder cancer, brain cancer, cervical cancer, CRC, ESCA, GC, HNC, liver cancer, lymphoma, melanoma, pancreatic cancer and PCa, as so as KIRP, READ, STAD, THCA, UCEC, CHOL, COAD, LUAD, and LUSC compared to the normal tissues. However, the results from Oncomine for breast, kidney, leukemia, and sarcoma cancer in different databases were opposite. The distinct levels of SPP1 expression in these cancers may be due to different data collection methods, which need further verification.

Subsequently, this study investigated the SPP1 prediction significance for cancers by the K-M plotter and PrognoScan databases. Our results on K-M plotter suggested that, the high SPP1 level significantly related to the poorer prognosis in CESC, HNSC, LUAD, and LUSC. Our results on PrognoScan database revealed that, the up-regulated SPP1 level showed inverse correlation with the OS for patients with BRCA, COAD, LUAD, and LUSC. However, there are some cancers such as LIHC, READ, and PAAD showed better prognosis when SPP1 was highly expressed. It suggested that SPP1 may possess the characteristics of oncogenes or anti-oncogenes, which was determined by the cancer type. These were the basis for further downstream analyses of SPP1. Whatever, this finding is the first report indicating SPP1 as the predictor to independently predict OS and RFS for pan-cancer.

For better exploring the SPP1 mechanism in promoting tumor progression and poor prognosis, we investigated and identified somatic mutations and CNV in COAD, HNSC, LUAD, and LUSC. Some important gene mutations such as TTN mutation in COAD and LUAD, LRP1B mutation in HNSC, TP53 mutation in LUAD were found (Supplementary Figure S1). However, only three common mutated genes (PLD5, DTX4, and USP25) were in the high SPP1 expression group. There was no common mutated gene in the group with low SPP1 expression, possibly because different tumors have different gene mutation profiles. These common mutant genes could not explain the function of SPP1 since they had a barely significant effect on prognosis and tumor progression.

Next, we identified SPP1 correlated common genes in four selected cancers. A total of 134 common genes were identified in three or more cancers. GO and KEGG analyses revealed that 134 common correlated genes were mainly enriched with extracellular structure organization, extracellular matrix organization, immune cells such as myeloid leukocyte, mononuclear and macrophage migration, activation, and positive regulation. Since myeloid leukocyte, mononuclear, and macrophages are enrolled into tumor microenvironment (TME) and activated as the tumor-associated cells, contributing to cancer growth and metastasis (Grivennikov et al., 2010). It is reasonable to believe that the effects of SPP1 on these common genes promote immune infiltration and cancer progression.

The correlation between SPP1 expression and miRNAs expression was analyzed based on TCGA database. We found two miRNAs were common in four cancers, and 19 miRNAs were common in three cancers. The miRNA-gene regulatory network was composed of 21 common miRNAs and 71 genes and displayed high connectivity interactions. Many of the 22 common genes and two common miRNAs found in this study have been extensively investigated. Moreover, the miRNA-gene regulation network revealed that CTSB was the target gene of hsa-miR-30c-5p, and MSR1 was the target gene of hsa-miR-152-3p. It is reported that CTSB could influence the invasive activity of lung cancer and shows significant association with the development of oral squamous cell carcinoma (OSCC) and predicts an increased overall mortality risk of colon cancer (Chan et al., 2010; Chen et al., 2012; Gong et al., 2013). Expressions of MMP12 and SULF1 were associated with tumor progression or metastasis in COAD, HNSC, LUAD, and LUSC (Lai et al., 2004; Qu et al., 2011; Kim et al., 2013; Klupp et al., 2016; Xie et al., 2018; Wei et al., 2020). Multiple studies have shown that ADAM12 is contributed to tumor progression and metastasis in COAD, HNSC, and LUAD (Mino et al., 2009; Rao et al., 2012; Ieguchi et al., 2014; Mochizuki et al., 2020). CD163 is mainly identified to be the unique monocyte/macrophage biomarker for M2 macrophages, and is tightly related to the dismal prognosis and aggressive phenotype of CRC, LUAD, LUSC, and OSCC (Cao et al., 2019; Li et al., 2019; Yang C. et al., 2019; Yang G. et al., 2019). This indicated the vital part of SPP1 in these cancers by interacting with these genes and miRNAs.

In cancer, TAMs and M2 macrophages, neutrophils, and DCs are key components of the tumor microenvironment (Lewis and Pollard, 2006; Giovanelli et al., 2019; Jaillon et al., 2020). The recent findings have suggested the vital parts of TIICs (including monocytes, neutrophils and macrophages) during tumor cell proliferation, metastasis or invasion to local and distant sites (Lewis and Pollard, 2006; Sawant et al., 2012; Szulzewsky et al., 2018). Our study showed that SPP1 facilitated immune cell infiltration, including macrophages, neutrophils, and DCs in COAD, HNSC, LUAD, and LUSC. Therefore, we believe that SPP1 may cause changes within TME.

Moreover, the association of SPP1 level with the immune cell marker sets implicated that SPP1 was involved in the regulation of tumor immunity. Recent results demonstrate the association of monocytes with tumor metastases and poor chemotherapeutic efficacy (Wang et al., 2019; Brunetti et al., 2020). Neutrophils exert the vital parts in cancer metastasis and progression (Swierczak et al., 2015). In this report, we found CD86, CD115, and CD11b were closely related to SPP1 levels in the context of COAD, HNSC, and LUAD. These results further confirmed that SPP1 plays a vital part in the polarization of monocytes and neutrophils, cancer progression and cancer metastasis. TAMs and M2 can promote tumor progression and metastasis (van Dalen et al., 2018; Wu et al., 2020). These findings disclosed that the up-regulated SPP1 level showed significant association with important markers of TAMs and M2 such as CCL2, CD68, IL10, CD163, VSIG4, and MS4A4A. TIM-3 (HAVCR2) could be detected in NSCLC, colorectal cancer and HNSC, and served as the predictor to independently predict LC and CRC (Jie et al., 2013; Du et al., 2017; Yu et al., 2017). SPP1 level was significantly correlated with TIM-3 (HAVCR2) in our study. These suggested that SPP1 exerts a vital part in TAMs, M2, and TIM-3 polarization and promoting tumor progression and metastasis in COAD, HNSC, LUAD, and LUSC. Interestingly, the correlation between SPP1 and VSIG4, MS4A4A, and HAVCR2 was consistent with the results from the TCGA databases. This further confirmed that SPP1 might promote tumor progress through interacting with common genes and facilitating immune cell infiltration in these cancers. However, further clinical investigation and basic experiments are needed to validate our results.

Intensive research on contribution of SPP1 in pathophysiology of cancer has unveiled the multifaceted role of SPP1 in tumor progression. Recent study reported that SPP1 can increase CD44 expression in prostate cancer cells (Bandopadhyay et al., 2014). Zou et al. (2011) have reported that SPP1 interacts with ITGB1 increase mesenchymal stem cells (MSCs) motility resulting in the enhanced metastasis and migration of cancer cells (Orian-Rousseau, 2010). In NSCLC, SPP1 induces vascular endothelial growth factor (VEGF) expression while facilitating disease progression (Lin et al., 2015). In colorectal cell, SPP1 negatively regulates T cell activation by bonding to CD44 and promote cancer progression (Shurin, 2018; Supplementary Figure S2A). Our results consistent with these reports and indicated that SPP1 has a more important correlation with CD44 and ITGB1 in selected cancer contrast to adjacent normal tissue. Therefore, it is reasonable to believe that SPP1 promotes tumor progression by interacting with the receptor in COAD, HNSC, LUAD, and LUSC. This work shed novel lights on the SPP1 function and receptor interactions in tumor immune infiltration. However, further clinical trials are needed to confirm this hypothesis.

Recent studies have reported that OPN/SPP1 has three splice variants (including OPN-a/b/c), which shows different features among diverse cancers (He et al., 2006; Supplementary Figure S2B). For example, overexpression of OPN-a in NSCLC displays the aggressive phenotype, whereas OPN-c presents the comparatively indolent phenotype (Goparaju et al., 2010). Moreover, OPN-c expression is detected in the invasive BCs, which shows high correlation with the survival outcomes for HER-2 BC cases (Mirza et al., 2008). It is important to understand the effect of individual variants, so as to adjust the suitable treatments for targeting OPN/SPP1. Unfortunately, in this study, we failed to obtain SPP1 splice variants data to further investigate their relationship with tumor-associated genes and immune infiltration.

In summary, our data revealed a previously uncharacterized mechanism that increased SPP1 expression correlated with poor prognosis and promoted tumor progression. Clinical trials based on SPP1 are conducted for evaluating the prognostic outcome and treatment response of cancer, which may provide guidance for the development of promising therapies. Targeting SPP1 and its receptor binding by specific blocking antibody is a feasible and effective way, and it may serve as the new immune-based treatment, especially in COAD, HNSC, LUAD, and LUSC.

## Data Availability Statement

The datasets presented in this study can be found in online repositories. The names of the repository/repositories and accession number(s) can be found in the article/Supplementary Material.

## Author Contributions

TW, GB, and YB conducted the statistical analysis and wrote the manuscript. SR, GY, HX, MZ, RS, YZ, and QW acquired the data and analyzed the data. YY and DZ designed the study and revised the manuscript. All authors contributed to the article and approved the submitted version.

## Conflict of Interest

The authors declare that the research was conducted in the absence of any commercial or financial relationships that could be construed as a potential conflict of interest.

## Footnotes

1 https://www.oncomine.org/resource/login.html

2 http://kmplot.com/analysis/

3 http://www.PrognoScan.org

4 https://cistrome.shinyapps.io/timer/

5 http://cancergenome.nih.gov/

6 http://zmf.umm.uni-heidelberg.de/apps/zmf/mirwalk2/

7 http://bioinformatics.psb.ugent.be/webtools/Venn/

8 https://www.ncbi.nlm.nih.gov/geo/

9 http://cibersort.stanford.edu/

10 http://gepia.cancer-pku.cn/index.html

11 https://string-db.org/
