# Supplementary figures and images for: The Significance of Secreted Phosphoprotein 1 in Multiple Human Cancers
Source: Front Mol Biosci. 2020 Nov 24;7:565383. doi: 10.3389/fmolb.2020.565383 (PMC7724571; doi:10.3389/fmolb.2020.565383)

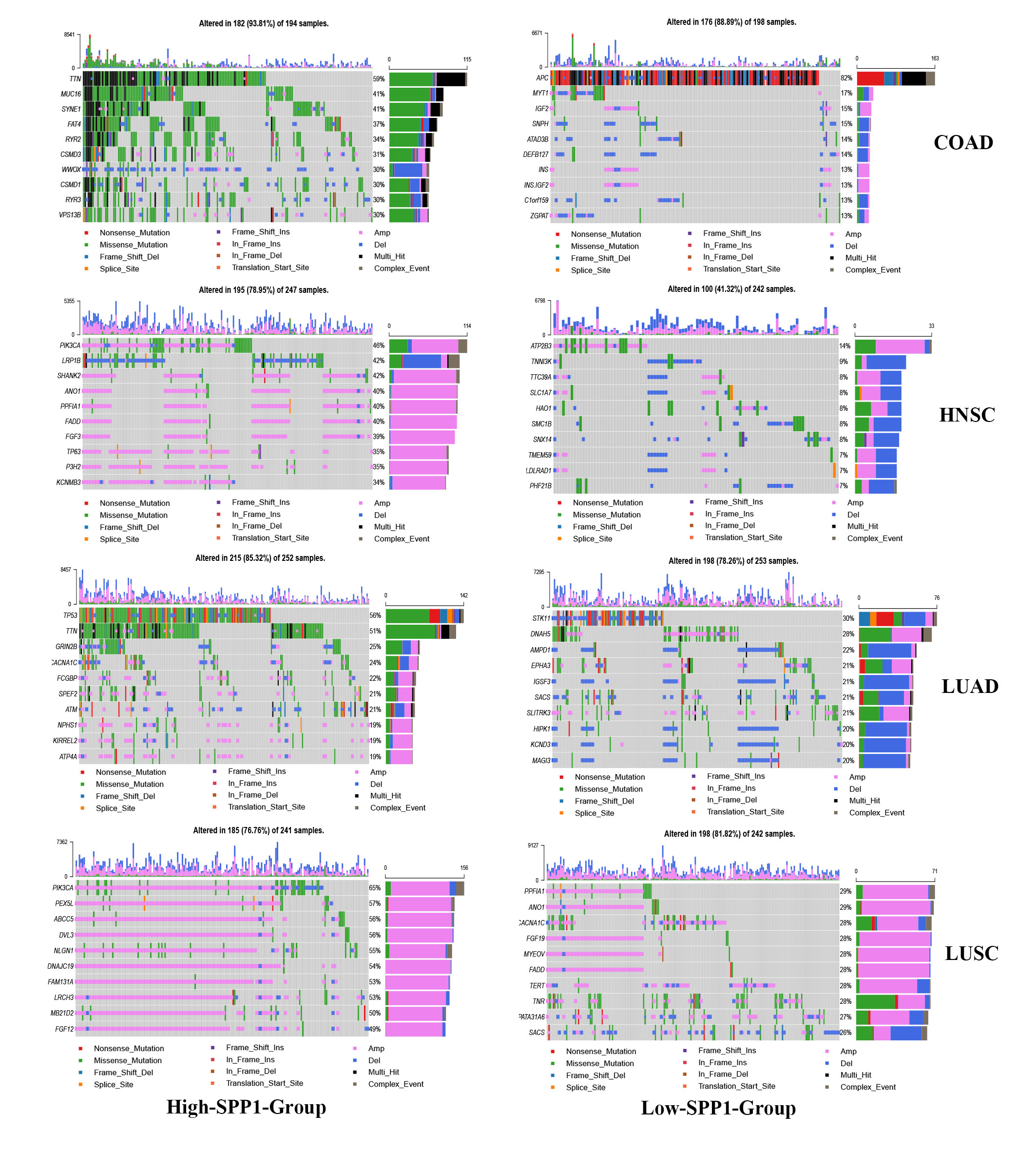

Supplement: Supplementary file 1 [file Image_1.JPEG]

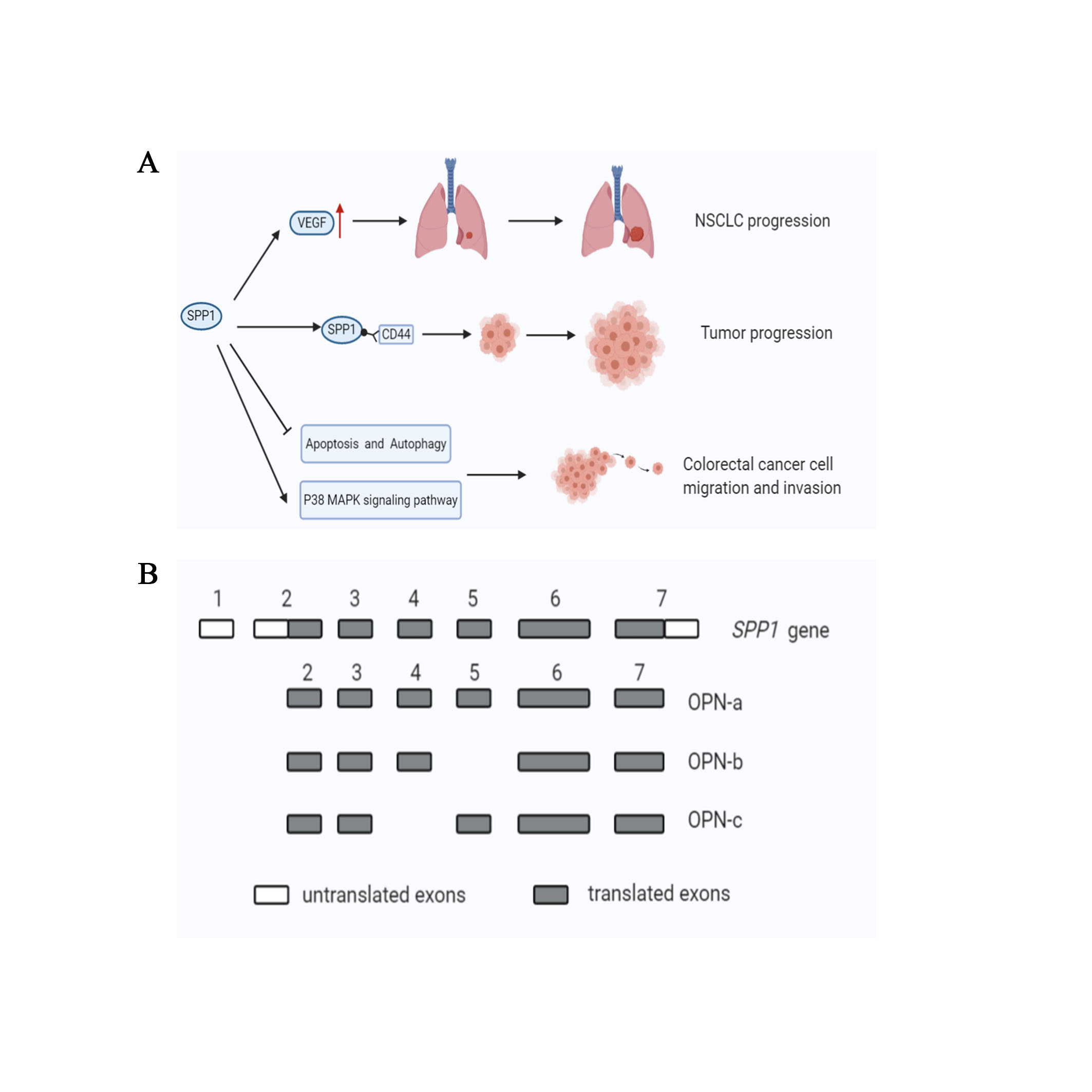

Supplement: Supplementary file 2 [file Image_2.JPEG]

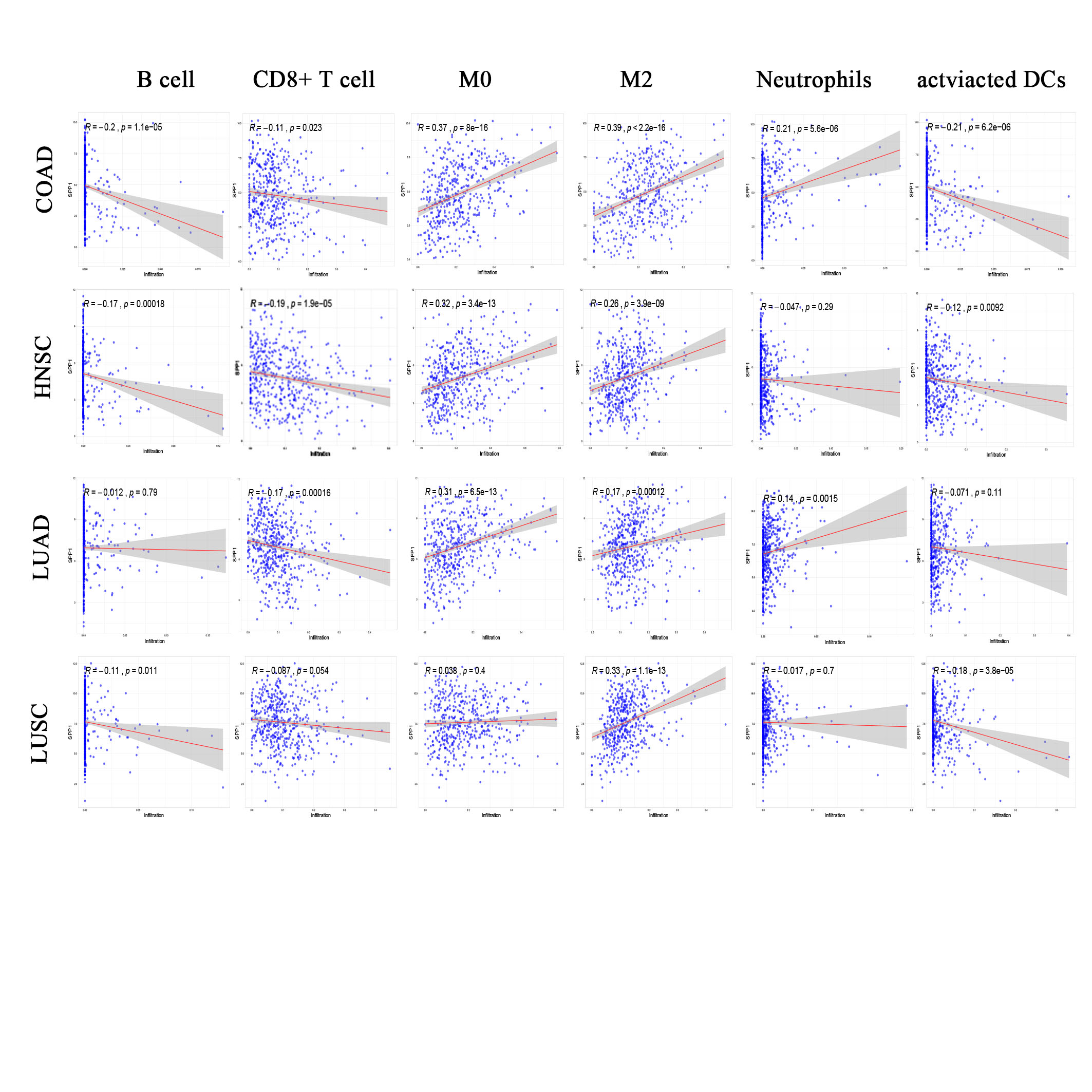

Supplement: Supplementary file 3 [file Image_3.JPEG]
