# Supplementary material for: The Significance of Secreted Phosphoprotein 1 in Multiple Human Cancers
Source: Front Mol Biosci. 2020 Nov 24;7:565383. doi: 10.3389/fmolb.2020.565383 (PMC7724571; doi:10.3389/fmolb.2020.565383)
Supplement: Supplementary file 4 [file Data_Sheet_1.docx]

**Supplementary Figure 1**| Tumor gene mutations associated to SPP1. The waterfall plots summarizing the gene mutations including somatic mutations and copy number variations in high and low SPP1 group. The type of alterations was annotated by different colors (bottom).

**Supplementary Figure 2**| The role of SPP1 in cancer progression and the structure of *SPP1* gene and SPP1 splice variant. **(A)** The role of SPP1 in cancer progression. **(B)** Structure of the *SPP1* gene and SPP1 splice variant. White boxes, untranslated exons; black boxes, translated exons. Osteopontin-a (OPN-a) represents the full-length cDNA, whereas osteopontin-b (OPN-b) has a deletion at translated exon 5, and osteopontin-c (OPN-c) has a deletion at translated exon 4.

**Supplementary Figure 3**| Analysis of the correlation between SPP1 and tumor‐infiltrating cells in CIBERSORT. COAD, colon adenocarcinoma (n=457); HNSC, head and neck cancer (n=457); LUAD, lung adenocarcinoma (n=515); LUSC, lung squamous cell carcinoma (n=507). B cell, B memory cells; M0, M0 macrophages; M2, M2 macrophages; activated DCs, activated dendritic cells.
