# Supplementary material for: The Significance of Secreted Phosphoprotein 1 in Multiple Human Cancers
Source: Front Mol Biosci. 2020 Nov 24;7:565383. doi: 10.3389/fmolb.2020.565383 (PMC7724571; doi:10.3389/fmolb.2020.565383)
Supplement: Supplementary file 5 [file Data_Sheet_2.docx]

Cancer is one of the leading causes of mortality worldwide and its incidence is growing rapidly. Although multimodal treatment strategies have evolved over the past few decades, the outcomes of cancer remain unsatisfactory, which is partly due to the scarcity of effective biomarkers for early detection and prognosis monitoring. Secreted phosphoprotein 1 (SPP1), also called Osteopontin (OPN), overexpressed in many cancers. However, the specific role of SPP1 in prognosis, gene mutations, and changes in gene and miRNA expression in human cancers is unclear. We systematically assessed SPP1 expression and its prognosis value in various cancers. Moreover, we evaluated the correlation between SPP1 and gene mutation, gene and miRNA expression in four selected tumors. SPP1-associated genes and miRNAs regulatory networks suggested that their interactions may play a role in the progression cancers. Then we investigated the influences of SPP1 on immune infiltration. These data provide strong evidence that SPP1 promote tumor progress through interacting with carcinogenic genes and facilitating immune cells’ infiltration. We believe that SPP1 may play a greater role in future immunotherapy, and the use of specific antibodies to block SPP1 and its receptor binding maybe a feasible and effective way.
